# Esophageal Squamous Cell Carcinoma and Gastric Cardia Adenocarcinoma Shared Susceptibility Locus in *C20orf54*: Evidence from Published Studies

**DOI:** 10.1038/srep11961

**Published:** 2015-07-08

**Authors:** Fujiao Duan, Shuli Cui, Chunhua Song, Xia Zhao, Liping Dai, Yong Shen

**Affiliations:** 1 Department of Hospital Infection Management, Affiliated Cancer Hospital of Zhengzhou University, Henan Cancer Hospital, Zhengzhou, Henan, China; 2 College of Professional Study, Northeastern University, Boston, Massachusetts, USA; 3 Department of Epidemiology, College of Public Health, Zhengzhou University, Zhengzhou, Henan, China; 4 Department of Clinical Laboratory, Affiliated Cancer Hospital of Zhengzhou University, Henan Cancer Hospital, Zhengzhou, Henan, China

## Abstract

This study aimed to determine whether *C20orf54* rs13042395 polymorphism modify the risk of esophageal squamous cell carcinoma (ESCC) and gastric cardia adenocarcinomas (GCA) in common population. We conducted a systematic literature review and evaluated the quality of included studies based on Newcastle-Ottawa Scale (NOS). Pooled odds ratios (ORs) and corresponding 95% confidence intervals (95%CIs) were calculated to estimate the strengths of the associations. 9 articles (10 studies) were identified for synthesis analyses. Overall, the results indicated that the *C20orf54* rs13042395 genotype was subtly decrease the risk of ESCC (T vs. C: OR = 0.95; 95%CI = 0.90–0.99; *P* = 0.02) and the rs13042395 polymorphism was associated with a decreased risk of GCA (T vs. C: OR = 0.95; 95%CI = 0.91–0.98; *P* < 0.01). The subsets were divided by smoking and drinking status, but none of the genetic comparisons reached statistical significance. Subgroup analysis was also stratified by body mass index (BMI), rs13042395 polymorphism was significantly associated with a subtly decreased cancer risk in under-weight group and normal group, but no association was observed in over-weight group. In conclusion, *C20orf54* rs13042395 polymorphism was significantly associated with decreased ESCC and GCA risk especially for the subjects with under-weight or normal.

Esophageal cancer and gastric cancer cause more than 400,000 and 700,000 deaths each year, respectively, and represent the sixth and second leading causes of cancer-related death worldwide^1^. Esophageal squamous cell carcinoma (ESCC) is the most frequent histological subtype of esophageal cancer and accounts for 90% of cases^2^,^3^. Synthesis of epidemiological studies indicate that alcohol drinking and tobacco smoking are the major risk factors for esophageal cancer^4^. *Helicobacter pylori* (*H.pylori*) infection is a well-established risk factor for Gastric Cardia Adenocarcinoma (GCA) and has been labeled as a definite human carcinogen by International Agency of Research on Cancer (IARC)^5^. In western countries, the high body mass index (BMI) has been suggested as a risk factor for GCA and esophageal adenocarcinoma^6^,^7^. However, only a subset of individuals exposed to the environmental risk factors would develop ESCC and GCA, it is suggested genetic factors substantially contribute to the ESCC and GCA carcinogenesis^8^.

In 2010, a large-scale genome-wide association study (GWAS) reported that a new and notable susceptibility locus (rs13042395) located in 5’ flanking region of chromosome 20 open reading frame 54 (C20orf54), it encodes riboflavin transporter 2 protein (RFT2) that was newly identified to play an important role in esophageal and carcinogenesis by modulating riboflavin uptake^9^. In addition, it has important biological implications for both ESCC and GCA in the Chinese population^10^,^11^. C20orf54 is a human riboflavin transporter that has an important role in the intestinal absorption of riboflavin^12^,^13^. The deficiency of riboflavin has been documented as a risk factor for ESCC and GCA. Also, riboflavin supplementation has been reported to reduce the risk of ESCC and GCA^14^.

For C20orf54 rs13042395 genotype and risk of ESCC and GCA, the results were inconsistent. On the basis of the biological and pathologic significance of *C20orf54*, it is widely shared that functional genetic variations in the *C20orf54* may contribute to the development of ESCC and GCA. The objective of the present study was to quantitatively assess the association between *C20orf54* rs13042395 polymorphism and risk of ESCC and/or GCA.

## Results

### Literature search and study characteristics

**Figure 1:**
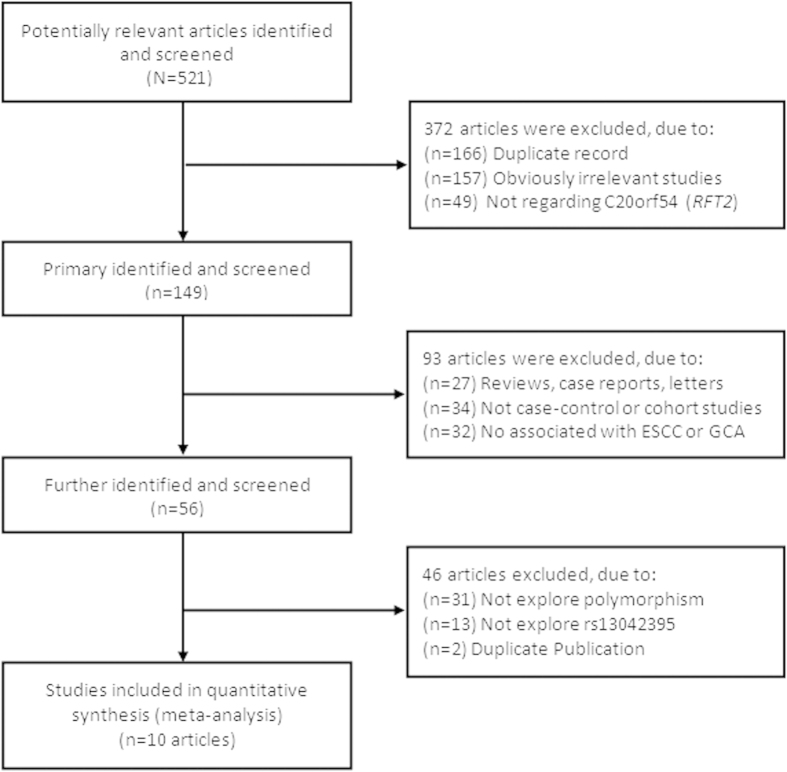
Flow diagram for screening and identification of relevant studies.

The selection process for relevant studies and a flow diagram are shown in Fig. 1. A computer-assisted search yielded 521 potentially relevant published titles. After primary identified, 149 titles were potentially appropriate, and the corresponding abstracts were reviewed. After further identification and screening individual study, 56 publications underwent full-text review. Finally, producing a total of 10 publications^10^,^15^,^16^,^17^,^18^,^19^,^20^,^21^,^22^,^23^ (12 studies) for inclusion. Characteristics of included studies are present in Table 1. We identified 12 studies, with a total of 88,547 participants, including 28,765 cases and 59,782 controls. The evidence synthesis included eight studies on ESCC, four GCA. There were 11 studies of Asian and one study of Caucasian. Of the 12 studies, 11 were population-based case-control studies and one was hospital-based case-control study, and eight studies were randomly repeated a portion of samples as quality control while genotyping.

### Assessment of methodological quality

The methodological quality assessment for included studies was summarized in Table 2. According to the NOS, Out of a maximum 9-point score, 4 studies had quality scores of 5–6, 8 studies had high quality scores of 7 or 8. The average scores of case-control studies were 6.67.

### Evidence synthesis

**Figure 2:**
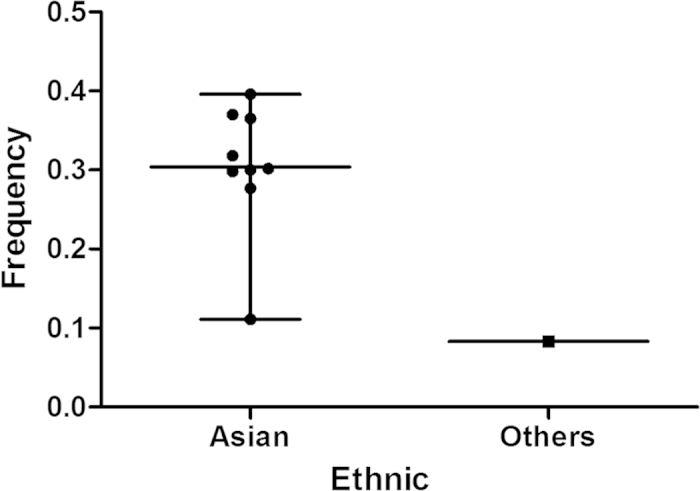
Frequencies of T allele in rs13042395 among controls stratified by ethnicity.

For all of 12 data sets, the frequencies of risk T allele in rs13042395 are presented in Fig. 2. The T allele frequencies for Asians and other populations were 30.41% and 8.30%, respectively.

**Figure 3:**
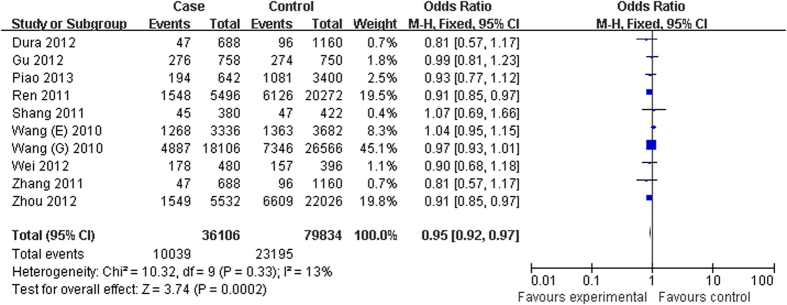
Forest plot of cancer risk associated with *C20orf54* rs13042395 for the allele comparison (T vs. C).

The evaluation of the association between the *C20orf54* rs13042395 polymorphism and the susceptibility to ESCC and GCA is presented in Table 3. Overall analysis indicated that the variant T allele of rs13042395 could significantly decrease the risk of ESCC and/or GCA in all genetic models (T vs. C: OR = 0.95, 95% CI = 0.92–0.97, *P* < 0.01; CT vs. CC: OR = 0.94, 95% CI = 0.88–0.99, *P* = 0.04; TT vs. CC: OR = 0.91, 95% CI = 0.83–0.99, *P* = 0.04; CT + TT vs. CC: OR = 0.94, 95% CI = 0.89–0.99, *P* = 0.01) except recessive model (TT vs. CT + CC: OR = 0.93, 95% CI = 0.86–1.02, *P* = 0.12) (Fig. 3).

The association between *C20orf54* rs13042395 polymorphism and ESCC risk was explored in eight studies. The results indicated that the *C20orf54* rs13042395 genotype subtly decreased the risk of ESCC, as revealed by the allele genetic model (T vs. C: OR = 0.95, 95% CI = 0.90–0.99, *P* = 0.02) (Table 3). GCA was defined by tumor site in four studies. The *C20orf54* rs13042395 polymorphism was associated with a decreased risk of GCA (T vs. C: OR = 0.95, 95% CI = 0.91–0.98, *P* < 0.01) (Table 3).

We performed subgroup analyses stratified by smoking status, all the genetic comparisons did not reach statistical significance in smokers (T vs. C: OR = 0.98, 95% CI = 0.89–1.08, *P* = 0.73; CT vs. CC: OR = 0.90, 95% CI = 0.66–1.22, *P* = 0.49; TT vs. CC: OR = 1.00, 95% CI = 0.79–1.27, *P* = 0.99; CT + TT vs. CC: OR = 0.96, 95% CI = 0.85–1.08, *P* = 0.48; TT vs. CT + CC: OR = 1.06, 95% CI = 0.84–1.33, *P* = 0.62) and never smokers (T vs. C: OR = 1.00, 95% CI = 0.94–1.06, *P* = 0.91; CT vs. CC: OR = 0.95, 95% CI = 0.87–1.02, *P* = 0.18; TT vs. CC: OR = 0.97, 95% CI = 0.84–1.11, *P* = 0.66; CT + TT vs. CC: OR = 0.94, 95% CI = 0.87–1.02, *P* = 0.12; TT vs. CT + CC: OR = 1.13, 95% CI = 0.76–1.67, *P* = 0.54) (Table 4).

In the subsets divided by drinking status, whereas no significant associations were detected among the drinkers (T vs. C: OR = 0.98, 95% CI = 0.88–1.10, *P* = 0.19; CT vs. CC: OR = 0.83, 95% CI = 0.56–1.22, *P* = 0.34; TT vs. CC: OR = 0.99, 95% CI = 0.75–1.31, *P* = 0.96; CT + TT vs. CC: OR = 0.95, 95% CI = 0.82–1.10, *P* = 0.51; TT vs. CT + CC: OR = 1.13, 95% CI = 0.76–1.67, *P* = 0.54) and never drinkers (T vs. C: OR = 0.96, 95% CI = 0.90–1.01, *P* = 0.11; CT vs. CC: OR = 0.95, 95% CI = 0.88–1.02, *P* = 0.17; TT vs. CC: OR = 0.92, 95% CI = 0.81–1.05, *P* = 0.22; CT + TT vs. CC: OR = 0.94, 95% CI = 0.88–1.01, *P* = 0.11; TT vs. CT + CC: OR = 0.94, 95% CI = 0.83–1.07, *P* = 0.39) (Table 4).

Furthermore, the subgroup analysis was stratified by BMI, *C20orf54* rs13042395 polymorphism was significantly associated with a subtly decreased cancer risk in under-weight group (T vs. C: OR = 0.87, 95% CI = 0.77–0.98, *P* = 0.02; TT vs. CC: OR = 0.70, 95% CI = 0.52–0.93, *P* = 0.02; TT vs. CT + CC: OR = 0.74, 95% CI = 0.56–0.98, *P* = 0.04) and normal weight group (T vs. C: OR = 0.85, 95% CI = 0.80–0.91, *P* < 0.01; TC vs. CC: OR = 0.82, 95% CI = 0.75–0.89, *P* < 0.01; CT + TT vs. CC: OR = 0.81, 95% CI = 0.75–0.88, *P* < 0.01), but no association was observed in over weight group (Table 4).

### Test of heterogeneity and sensitivity analysis

Our data sets indicated that there was no significant heterogeneity between studies among all comparisons in the overall analysis (*P*_heterogeneity_ > 0.05, *I*^*2*^ ≦ 50%). One-way sensitivity analyses were performed to assess the influence of the results by the systematic omission of the individual studies from the analyses. The dataset showed that the corresponding pooled ORs were not materially altered, indicating that our results were statistically robust (data not shown).

### Publication bias

**Figure 4:**
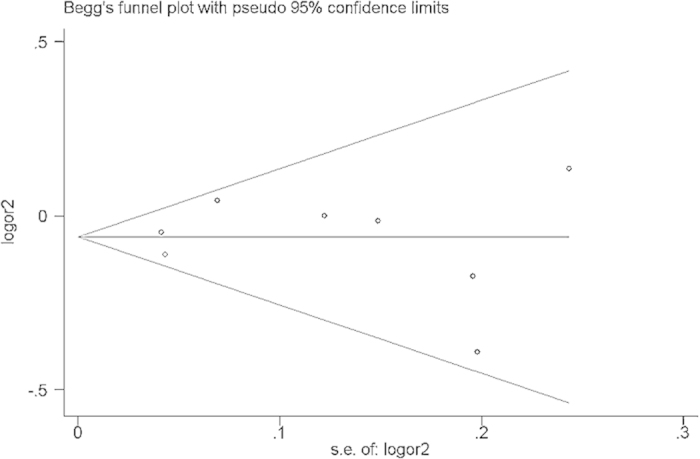
Funnel plot of *C20orf54* rs13042395 polymorphism and susceptibility to ESCC and GCA for dominant model (CT + TT vs. CC).

There was no evidence for publication bias using either Begg’s rank correction. Begg’s funnel plot and Egger’s linear regression test were performed to assess the publication bias of the quantitative synthesis literature. The shape of the funnel plot (Begg’s rank correction) did not reveal any evidence of obvious asymmetry (Fig. 4), and no evidence for publication bias using Egger’s linear regression test (Table 5).

## Discussion

Results from previous individual published studies investigating the associations between *C20orf54* rs13042395 polymorphism and cancer risk (ESCC and/or GCA) were inconclusive. The present study is considered to be the first quantitative meta-analysis concerning the effect of *C20orf54* rs13042395 polymorphism on risks of ESCC and GCA and specific stratified analysis (smoking status, drinking status and BMI). By analyzing the data that extracted from 10 published studies, we revealed that *C20orf54* rs13042395 polymorphism might be associated with decreased ESCC and GCA risk especially for under-weight and normal weight groups.

The genetic basis of ESCC and GCA between a large number of SNPs and disease predisposition has been explored, and the rs13042395 in *C20orf54* was significantly associated with ESCC and GCA risk in the GWAS among Chinese population^10^. However, other two Chinese population-based GWASs both failed to expore a significant association of rs13042395 with the risk of ESCC and GCA^2^,^11^. In the present study, we identified a significant association of rs13042395 with the risk of ESCC and GCA. This indicated that the finding of GWAS need independent replication studies to verify.

ESCC and GCA are complex diseases likely resulting from multiple interacting genetic polymorphisms and gene-environment interactions. Both in the western countries and Asian especially in China, heavy smoking and alcohol consumption were identified as the main environmental risk factors for ESCC and GCA^24^,^25^. C20orf54 has a high homology with rat C20orf54, a transmembrane protein involved in the uptake of riboflavin in the small intestine^10^. The *C20orf54* genotypes modulated the risk of ESCC in smokers, drinkers, or in individuals with a negative family history^18^. These findings suggest that C20orf54 may alter environmental risk factors. Interestingly, our results indicated that smoking and drinking did not significantly alter the effects of *C20orf54* rs13042395 polymorphism on the risk of ESCC and/or GCA. However, on this point, our meta-analysis obtained the consistent conclusions came up with Wang *et al*.^10^.

In the present study, the *C20orf54* rs13042395 T allele significantly decreased the risk of ESCC and/or GCA in the subjects with BMI less than 24 especially between 18.5 to 24. Overweight and obesity have been consistently related to gastric and esophageal adenocarcinoma, but not to squamous cell carcinoma^26^,^27^,^28^. The influence of obesity on gastric and esophageal adenocarcinoma may be related to higher incidence of gastroesophageal reflux in obese individuals^29^, and the risk of gastroesophageal reflux is strongly associated with the risk for Barrett’s esophagus^30^,^31^.

The following limitations should be acknowledged in our studies. First, the present meta-analysis only included design of case-control studies, some of which were hospital based studies. Thus, the controls may not reflect the representative element of the source population. Second, although all eligible studies were summarized, the relatively small study number may lead to reduced statistical power when stratified according to the cancer type, ethnicity, smoking status, drinking status and BMI. Third, the pooled datasets without excluding the studies with inefficient points based on NOS. In addition, Large-scale studies will be needed for high-risk population screening, individualized prevention, treatment and exposure rating in the future.

In summary, current data suggest that *C20orf54* rs13042395may be associated with a significantly decreased risk of ESCC and GCA, especially for the subjects with BMI less than 24 particularly between 18.5 to 24. Notably, based on the well-designed studies at multicenters with large sample size will be needed for further validate our results.

## Materials and Methods

### Data source and search strategy

We comprehensively identified studies through searching PubMed, Embase, Web of Science, Chinese National Knowledge Infrastructure (CNKI) and Wanfang database using terms “*C20orf54*”, “*RFT2*” and “rs13042395” for both case–control and cohort studies, which evaluated the association between *C20orf54* rs13042395 polymorphism and the risk of ESCC and/or GCA (last search update: March 24, 2015). The search was limited to papers published in English or Chinese language. In addition, Reference lists of retrieved articles were examined manually to further identify potentially relevant studies.

### Inclusion and exclusion criteria

Studies were included in the analysis if following criteria were met: (i) based on case-control studies (including cohort studies and GWASs) examined the associations between the *C20orf54* rs13042395 and ESCC or GCA; (ii) sufficient allele or genotype data for estimating an odds ratio (OR) with corresponding 95% confidence intervals (95%CIs); (iii) genotype distribution of control groups must be in accordance with the assumptions of Hardy-Weinberg equilibrium (HWE). Case-control studies based on the esophageal adenocarcinomas and/or gastric non-cardia adenocarcinoma were excluded. In case of redundant publications, only the studies with the largest sample size and/or latest published date were included.

### Data extraction and quality assessment

Two independent authors (Fujiao Duan and Shuli Cui) extracted the data from the eligible publications. Data for analyses, including first author, publication year, study design, ethnicity, cancer type, source of control, detection methods of *C20orf54* rs13042395 polymorphism and quality control or not, characteristics of cases and controls. If discrepancies existed, consensus would be finally reached on discussion.

We assessed quality of included studies by a modified checklist based on the Newcastle–Ottawa Scale (NOS)^32^, with discrepancies resolved by consensus. A nine-point scale of the NOS (range, 0–9 points) has been developed for the evaluation. A high-quality study was defined as one with great than or equal to 7 points.

### Quantitative data synthesis and analyses

We utilized RevMan 5.0 (Cochrane Collaboration, Oxford, UK) and STATA 12.0 (StataCorp, College Station, TX, USA) to perform all the statistical analysis.

RevMan 5.0 was used to estimate the association between *C20orf54* rs13042395 polymorphism and cancer risk by the pooled ORs with corresponding to 95%CIs. The stratified analysis was conducted by ethnicity (Asian, Caucasian), smoking status (smokers, never smokers), drinking status (drinkers, never drinkers) and BMI (under weight <18.5, normal weight: 18.5–24, over weight >24).

Heterogeneity was explored by the chi-squared test (*χ*^*2*^) of heterogeneity and the inconsistency index (*I*^*2*^) between each individual study. By heterogeneity test, if *P*-value for heterogeneity test (*P*_heterogeneity_) < 0.05 or *I*^2^ > 50%, the sources of heterogeneity would be used for meta regression in STATA 12.0^33^. Random- or fixed-effects models were used depending on *P*_heterogeneity_. If *P*_heterogeneity_ ≥ 0.05, we used the fixed effect model (the Mantel-Haenszel method)^34^. Otherwise, random effects model (DerSimonian and Laird method) was selected^35^. The significance of merged OR was dependent on the Z-test, *P* < 0.05 was considered significant.

Sensitivity analysis, in which one study is omitted at a time, was performed to assess the quality and consistency of the results.

Publication bias was evaluated by Begg’s test (rank correlation test)^36^ and then statistically using Egger’s test (weighted linear regression test)^37^. This analysis was performed using the STATA 12.0 procedure of ‘Metabias’.

## Additional Information

**How to cite this article**: Duan, F. *et al*. Esophageal Squamous Cell Carcinoma and Gastric Cardia Adenocarcinoma Shared Susceptibility Locus in *C20orf54*: Evidence from Published Studies. *Sci. Rep.* **5**, 11961; doi: 10.1038/srep11961 (2015).

## Figures and Tables

**Table 1:** Characteristics of included studies.

Author	Year	Study type	Ethnicity (Country)	Cancer type	Source of control	Genotyping	Matching Y/N	Sample size Case/Control	Quality^a^ control	*P*_HWE_
Peng[15]	2014	Case-control	Asian (China)	ESCC	Population	Sequenom	N	50/50	N	NA
Peng[15]	2014	Case-control	Asian (China)	GCA	Population	Sequenom	N	50/50	N	NA
Piao [16]	2014	Case-control	Asian (Korea)	ESCC	Population	HRM	Y	321/1700	Y	0.724
Gu [17]	2012	Case-control	Asian (China)	ESCC	Hospital	MassArray	Y	379/375	Y	0.648
Wei [18]	2012	Case-control	Asian (China)	ESCC	Population	PCR-RFLP	Y	240/198	Y	0.354
Dura [19]	2012	Case-control	Caucasian (Netherland)	ESCC	Population	Real-Time PCR	Y	344/580	N	0.267
Zhou [20]	2012	Case-control	Asian (China)	ESCC	Population	Chips	Y	4722/4732	Y	0.639
Ren [21]	2011	Case-control	Asian (China)	GCA	Population	Chips	Y	2748/10136	Y	0.407
Shang [22]	2011	Case-control	Asian (China)	ESCC	Population	MALDI-TOF-MS	Y	190/211	N	0.336
Zhang [23]	2011	Case-control	Asian (China)	GCA	Population	TaqMan	Y	1668/1841	Y	0.284
Wang [10]	2010	GWAS	Asian (China)	ESCC	Population	Chips	Y	9053/13283	Y	NA
Wang [10]	2010	GWAS	Asian (China)	GCA	Population	Chips	Y	2766/11013	Y	NA

ESCC, Esophageal squamous cell carcinoma; GCA, Gastric cardia adenocarcinoma; NA: Not applicable for the lack of C allele;

HRM, High-resolution melting; MALDI-TOF-MS, Matrix-Assisted Laser Desorption/Ionization Time of Flight Mass Spectrometry;

^a^Quality control was conducted when sample of cases and controls was genotyped.

**Table 2:** Methodological quality of studies included in the meta-analysis.

	Selection (score)				Comparability (score)	Exposure (score)			
Study	Adequate definition of patient case	Representativeness of patients cases	Selection of controls	Definition of control	Control for important factor or additional factor	Ascertainment of exposure (blinding)	Same method of ascertainment for participants	Non-response Rate^a^	Total Score^b^
Peng[15]	1	1	1	1	0	0	1	0	**5**
Peng[15]	1	1	1	1	0	0	1	0	**5**
Piao [16]	1	1	1	1	2	0	1	1	**8**
Gu [17]	1	1	0	1	2	0	1	0	**6**
Wei [18]	1	1	1	1	2	0	1	0	**7**
Dura [19]	1	1	1	1	2	0	1	0	**7**
Zhou[20]	1	1	1	1	2	0	1	0	**7**
Ren[21]	1	1	1	1	2	0	1	0	**7**
Shang [22]	1	1	1	1	1	0	1	0	**6**
Zhang [23]	1	1	1	1	2	0	1	1	**8**
Wang [10]	1	1	1	1	2	0	1	0	**7**
Wang[10]	1	1	1	1	2	0	1	0	**7**

^a^When there was no statistical significance in the response rate between case and control groups by using a chi-squared test (*P* > 0.05), one point was awarded;

^b^Total score was calculated by adding up the points awarded in each item.

**Table 3:** Main results of pooled ORs in the meta-analysis.

	Cases	Controls	Heterogeneity test				Hypothesis test		
Comparisons	*n/N*	*n/N*	*Q*	*P*	*I*^2^(%)	Summary OR (95% CI)	*Z*	*P*	Studies
Total									
T vs C	10039/36106	23195/79834	10.32	0.33	13	0.95(0.92,0.97)	3.74	<0.01	10
CT vs CC	4304/9515	8381/17945	9.68	0.21	28	0.94(0.88,0.99)	2.04	0.04	8
TT vs CC	953/6308	1820/11536	8.57	0.29	18	0.91(0.83,0.99)	2.08	0.04	8
CT + TT vs CC	5306/10712	10115/19865	9.25	0.41	3	0.94(0.89,0.99)	2.50	0.01	10
TT vs CT + CC	953/10612	1820/19765	9.92	0.19	29	0.93(0.86,1.02)	1.55	0.12	8
ESCC									
T vs C	8444/29922	16973/58402	6.90	0.33	13	0.95(0.90,0.99)	2.24	0.02	7
CT vs CC	2416/6140	2657/7147	7.21	0.21	31	0.95(0.89,1.03)	1.24	0.22	6
TT vs CC	489/3780	649/4706	3.21	0.67	0	0.91(0.80,1.04)	1.38	0.17	6
CT + TT vs CC	2931/6246	3768/7846	4.38	0.63	0	0.95(0.88,1.02)	0.49	0.14	7
TT vs CT + CC	489/6196	649/7796	5.87	0.32	15	0.94(0.83,1.07)	0.96	0.34	6
GCA									
T vs C	1595/6584	6222/21432	3.42	0.18	42	0.95(0.91,0.98)	3.00	<0.01	3
CT vs CC	1888/3375	5724/10798	2.25	0.13	55	0.93(0.85,1.01)	1.69	0.09	2
TT vs CC	464/2582	1171/6830	5.35	0.02	81	0.93(0.69,1.26)	0.45	0.65	2
CT + TT vs CC	2375/4466	6347/12019	4.75	0.09	58	0.93(0.87,1.00)	2.00	0.05	3
TT vs CT + CC	464/4416	1171/11969	4.02	0.05	75	0.93(0.82,1.05)	1.22	0.22	2

**Table 4:** Stratified analyses of the *C20orf54* rs13042395 polymorphism on cancer risk.

	Heterogeneity test				Hypothesis test		
Comparisons	*Q*	*P*	*I*^*2*^(%)	Summary OR (95%CI)	*Z*	*P*	Studies
Smoking status							
Smokers							
T vs C	0.95	0.81	0	0.98(0.89,1.08)	0.34	0.73	4
CT vs CC	11.38	0.01	74	0.90(0.66,1.22)	0.68	0.49	4
TT vs CC	1.92	0.59	0	1.00(0.79,1.27)	0.02	0.99	4
CT + TT vs CC	4.79	0.19	37	0.96(0.85,1.08)	0.70	0.48	4
TT vs CT + CC	5.62	0.13	47	1.06(0.84,1.33)	0.50	0.62	4
Never smokers							
T vs C	3.47	0.33	13	1.00(0.94,1.06)	0.11	0.91	4
CT vs CC	0.30	0.96	0	0.95(0.87,1.02)	0.35	0.18	4
TT vs CC	1.13	0.77	0	0.97(0.84,1.11)	0.44	0.66	4
CT + TT vs CC	0.52	0.91	0	0.94(0.87,1.02)	1.56	0.12	4
TT vs CT + CC	14.63	<0.01	79	1.13(0.76,1.67)	0.61	0.54	4
Drinking status							
Drinkers							
T vs C	0.36	0.95	0	0.98(0.88,1.10)	0.27	0.19	4
CT vs CC	12.86	0.01	77	0.83(0.56,1.22)	0.96	0.34	4
TT vs CC	0.59	0.90	0	0.99(0.75,1.31)	0.06	0.96	4
CT + TT vs CC	4.62	0.20	35	0.95(0.82,1.10)	0.65	0.51	4
TT vs CT + CC	4.75	0.19	37	1.07(0.82,1.40)	0.53	0.60	4
Never drinkers							
T vs C	1.02	0.80	0	0.96(0.90,1.01)	1.61	0.11	4
CT vs CC	0.88	0.83	0	0.95(0.88,1.02)	1.38	0.17	4
TT vs CC	0.67	0.88	0	0.92(0.81,1.05)	1.22	0.22	4
CT + TT vs CC	1.05	0.79	0	0.94(0.88,1.01)	1.59	0.11	4
TT vs CT + CC	0.43	0.93	0	0.94(0.83,1.07)	0.87	0.39	4
BMI							
Under weight							
T vs C	0.58	0.75	0	0.87(0.77,0.98)	2.26	0.02	3
CT vs CC	0.38	0.83	0	0.91(0.77,1.08)	1.06	0.29	3
TT vs CC	0.66	0.72	0	0.70(0.52,0.93)	2.42	0.02	3
CT + TT vs CC	1.95	0.38	0	0.89(0.76,1.05)	1.41	0.16	3
CC vs CT + TT	1.48	0.48	0	0.74(0.56,0.98)	2.07	0.04	3
Normal weight							
T vs C	0.69	0.71	0	0.85(0.80,0.91)	4.76	<0.01	3
CT vs CC	3.95	0.14	0	0.82(0.75,0.89)	4.49	<0.01	3
TT vs CC	0.74	0.69	0	0.76(0.69,0.85)	3.35	<0.01	3
CT + TT vs CC	1.49	0.47	0	0.81(0.75,0.88)	4.90	<0.01	3
CC vs CT + TT	0.55	0.76	0	0.93(0.74,1.16)	0.67	0.50	3
Brought forward							
Over weight							
T vs C	2.62	0.27	24	1.03(0.88,1.21)	0.38	0.70	3
CT vs CC	0.70	0.71	0	1.02(0.82,1.26)	0.19	0.85	3
TT vs CC	3.46	0.18	42	1.06(0.73,1.54)	0.31	0.76	3
CT + TT vs CC	1.17	0.56	0	1.04(0.85,127)	0.37	0.71	3
CC vs CT + TT	5.54	0.06	64	1.08(0.76,1.54)	0.45	0.66	3

**Table 5:** Publication bias of *C20orf54* rs13042395 for Egger’s test.

Comparisons	*t*	*p*	95% CI
T vs C	−0.54	0.604	−2.066 ~ 1.283
CT vs CC	−0.09	0.933	−1.995 ~ 1.857
TT vs CC	−0.71	0.505	−2.163 ~ 1.191
CT + TT vs CC	−0.01	0.993	−1.768 ~ 1.756
CC vs CT + TT	−0.52	0.619	−2.239 ~ 1.450
